# Efficient Collection and Representation of Preverbal Data in Typical and Atypical Development

**DOI:** 10.1007/s10919-020-00332-4

**Published:** 2020-03-02

**Authors:** Florian B. Pokorny, Katrin D. Bartl-Pokorny, Dajie Zhang, Peter B. Marschik, Dagmar Schuller, Björn W. Schuller

**Affiliations:** 1 iDN – interdisciplinary Developmental Neuroscience, Division of Phoniatrics, Medical University of Graz, Graz, Austria; 2 Machine Intelligence & Signal Processing group (MISP), Chair of Human–Machine Communication, Technical University of Munich, Munich, Germany; 3 Department of Child and Adolescent Psychiatry and Psychotherapy, University Medical Center Göttingen, Göttingen, Germany; 4 Leibniz ScienceCampus Primate Cognition, Göttingen, Germany; 5 Center of Neurodevelopmental Disorders (KIND), Department of Women’s and Children’s Health, Karolinska Institutet, Stockholm, Sweden; 6 audEERING GmbH, Gilching, Germany; 7 ZD.B Chair of Embedded Intelligence for Health Care and Wellbeing, University of Augsburg, Augsburg, Germany; 8 GLAM – Group on Language, Audio & Music, Department of Computing, Imperial College London, London, UK

**Keywords:** Preverbal development, Data collection, Data representation, Infancy, Developmental disorders, Intelligent audio analysis

## Abstract

Human preverbal development refers to the period of steadily increasing vocal capacities until the emergence of a child’s first meaningful words. Over the last decades, research has intensively focused on preverbal behavior in typical development. Preverbal vocal patterns have been phonetically classified and acoustically characterized. More recently, specific preverbal phenomena were discussed to play a role as early indicators of atypical development. Recent advancements in audio signal processing and machine learning have allowed for novel approaches in preverbal behavior analysis including automatic vocalization-based differentiation of typically and atypically developing individuals. In this paper, we give a methodological overview of current strategies for collecting and acoustically representing preverbal data for intelligent audio analysis paradigms. Efficiency in the context of data collection and data representation is discussed. Following current research trends, we set a special focus on challenges that arise when dealing with preverbal data of individuals with late detected developmental disorders, such as autism spectrum disorder or Rett syndrome.

## Introduction

Of all significant changes during infancy, the acquisition of verbal abilities is one of the most striking phenomena for parents, clinicians, and researchers. Several vocal transformations take place between a newborn’s first cry and the production of first meaningful words, usually around the end of the first year of life (e.g., Nathani et al. 2006; Oller 1980, 2000; Papoušek 1994; Stark 1980, 1981; Stark et al. 1993). Vocal patterns with salient characteristics emerging in this preverbal period are—amongst others—cooing around the third month post-term age (Nathani et al. 2006; Oller 1980; Stark 1980) and canonical babbling around the eighth month post-term age (Nathani et al. 2006; Oller 1980; Papoušek 1994; Stark 1980). Preverbal development is related to fundamental processes of infant brain development (e.g., Dehaene-Lambertz 2017) in combination with anatomical and voice-physiological changes (e.g., Holzki et al. 2018).

For almost 40 years, typical preverbal development has been intensively studied (e.g., Locke 1995; Oller 1980, 2000; Stark 1981; Stark et al. 1993). Besides seeking for appropriate schemes to phonetically categorize preverbal behavior (e.g., Nathani et al. 2006; Oller 1980; Papoušek 1994; Stark 1980, 1981), research has focussed on defining milestones of preverbal development, such as the above-mentioned onset of canonical babbling (Harold and Barlow 2013). Delays in reaching specific milestones or their non-achievement have been discussed as potential early indicators of atypical development (e.g., Lang et al. 2019; Lohmander et al. 2017; Oller et al. 1998). In fact, a number of developmental disorders are associated with deficits in the speech-language domain. Some of these disorders can be recognized at birth or even earlier. For example, infants with Down syndrome have a characteristic physical appearance and specific morphological features leading to clinical and genetic diagnosis (World Health Organization 2019). Other developmental disorders are lacking apparent early signs. These disorders are identified when certain physical features become apparent, developmental milestones are not achieved or their achievement is delayed, or behavioral/neurofunctional deviances reach a certain threshold to allow clinical diagnosis. The late clinical manifestation of these disorders currently leads to an accurate diagnosis of affected children usually not before toddlerhood (Baio et al. 2018; Christensen et al. 2016; Marschik et al. 2016; Sicherman et al. 2018; Tarquinio et al. 2015).

One of these best known late detected developmental disorders is autism spectrum disorder (ASD; American Psychiatric Association 2013; World Health Organization 2019). The current prevalence of ASD is 1 in 59 children in the USA (Baio et al. 2018) with a higher occurrence in males than in females (e.g., Baio et al. 2018), and a recurrence risk of up to 18% for younger siblings of children already diagnosed with ASD (e.g., Bhat et al. 2014; Bölte 2014). The exact etiology of ASD is still unknown (e.g., American Psychiatric Association 2013; Bhat et al. 2014; Bölte et al. 2019). Another late detected developmental disorder that is associated with deficits in the speech-language domain is Rett syndrome (RTT; World Health Organization 2019), occurring in about 1 in 10,000 live female births (Laurvick et al. 2006). De novo mutations in the X chromosome-linked gene *MECP2* were identified as its main cause (Amir et al. 1999). In most cases, affected male individuals do not survive the prenatal period (Tokaji et al. 2018). In both ASD and RTT, as well as in a number of other late detected developmental disorders, diagnosis criteria include deficits in the socio-communicative and speech-language domains (American Psychiatric Association 2013; Neul et al. 2010). Research is increasingly focusing on preverbal phenomena in these disorders in order to find early markers for an earlier identification of affected individuals. However, many of the existing studies were based upon limited datasets. For example, some individuals with RTT were found not to acquire certain preverbal capacities, such as cooing, babbling, or the production of proto-words (Bartl-Pokorny et al. 2013; Marschik et al. 2013, 2014a). The latter are word-like vocalizations that do not yet conform to target language concerning articulation and/or lexical meaning (Kauschke 2000; Papoušek 1994). The preverbal behavior of individuals with RTT was reported to have an intermittent character of apparently typical and atypical vocalization patterns, such as sequences of high-pitched crying-like phonation, phonation with an ingressive pulmonic airstream, or phonation with a pulmonic airstream of excessive pressure (e.g., Marschik et al. 2009, 2012a, 2013, 2014a; Pokorny et al. 2018). Repeatedly documented preverbal atypicalities in individuals with ASD are a late onset of canonical babbling, a low canonical babbling ratio, a comparably low rate of vocalization, and monotonous intonation patterns (e.g., Chericoni et al. 2016; Patten et al. 2014; Paul et al. 2011; Roche et al. 2018).

While the first intensive efforts of preverbal sound categorization were made in the 1980s (e.g., Oller 1980; Stark 1980, 1981), the first acoustic data representations were used to describe recorded preverbal behavior at a signal level. For example, investigations by Kent and Murray (1982) were based on small sets of extracted acoustic features, such as vocalization duration, variations of the vocal tract source excitation, or formant frequencies. Bloom et al. (1999) calculated frequency–amplitude slopes in preverbal syllabic sounds as a measure of nasality. In addition, the fundamental frequency (F0), i.e., the lowest frequency of vocal fold oscillation, has always been a frequently extracted feature—in voice analytics in general, but also for acoustic preverbal sound characterization (e.g., Keating and Buhr 1978; Kent and Murray 1982; Petroni et al. 1994; Robb et al. 1989). A number of recent studies on crying vocalizations also reported on F0-related preverbal atypicalities in individuals with ASD (e.g., Esposito and Venuti 2010; Esposito et al. 2013; Sheinkopf et al. 2012).

With the rising age of high-performance computing, the request for efficient preverbal behavior analysis has also grown. The discipline of intelligent audio analysis (Schuller 2013), which deals with the combination of advanced audio signal processing and machine learning technology, provided the methodological tools for the automatic retrieval of preverbal sound-related (meta-)information. Specific tasks are, e.g., the automatic differentiation of preverbal vocalization types (e.g., Schuller et al. 2019), the automatic detection of infant distress (e.g., Chang and Li 2016; Chang et al. 2015; Lavner et al. 2016; Rodriguez and Caluya 2017; Schuller et al. 2018), or the automatic recognition of the medical condition of the infant who produces the vocalization (e.g., Orlandi et al. 2012; Pokorny et al. 2016a, 2017). However, different learning tasks require different learning strategies. These involve different ‘optimal’ representations of the collected preverbal data.

Building on our experience in collecting and analyzing preverbal data of typical and atypical development (e.g., Bartl-Pokorny et al. 2013; Marschik et al. 2012a, b, 2013, 2014a, b, 2017; Pokorny et al. 2016a, 2017, 2018), we provide a methodological overview of current strategies for the collection and representation of preverbal data for intelligent audio analysis purposes. Exemplified on the basis of empirical data, we will especially focus on application-oriented challenges and constraints that have to be considered when dealing with preverbal data of individuals with late detected developmental disorders. Finally, data collection and representation of preverbal behavior will be discussed in context of efficiency.

## Data Collection

**Fig. 1:**
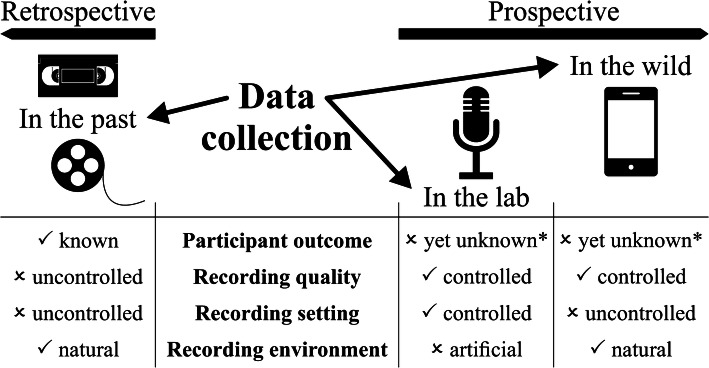
Overview of strategies for collecting preverbal data and strategy comparison on the basis of study design criteria chronologically referring to the period of data collection. *For typically developing participants or participants with a late detected developmental disorder

The process of data collection for acoustic studies on typical and atypical preverbal development involves the recording of preverbal behavior itself by means of a recording device and the acquisition of study-relevant meta-information, such as the participant’s chronological age at the time of recording and his or her medical history. In particular, the participant’s developmental outcome in terms of typical versus atypical has to be carefully documented. This, however, raises a crucial methodological issue for the collection of preverbal data of infants with a late detected developmental disorder. Dependent on a priori study criteria and potential future applications building on a study’s findings, three possible strategies for the collection of preverbal data can be distinguished: data collection (1) ‘in the past’, (2) in the lab, or (3) ‘in the wild’ (Fig. 1). Referring to a study’s timeline, these data collection strategies can be categorized as either retrospective or prospective approaches, respectively.

Prospective data collection can be carried out in the lab or in the participant‘s natural environment, here referred to as ‘in the wild’. Infants fulfilling specific inclusion criteria, can be systematically recruited and prospectively studied in a longitudinal study design with scheduled outcome assessments. Such procedures are well-suited for investigations in neurotypical cohorts, but also allow for the collection of preverbal data in late detected developmental disorders with known a priori risk factors, a high prevalence, and/or a high recurrence risk. This applies, for example, to ASD. Prospective high-risk ASD studies usually build upon the high familial recurrence of ASD (e.g., Bhat et al. 2014; Bölte 2014) and follow younger siblings of children with an existing ASD diagnosis. This is done in various research institutions and multi-center approaches mainly across Europe and the USA (e.g., Bölte et al. 2013; Ozonoff et al. 2011a).

A prospective collection of preverbal data of individuals with rare late detected developmental disorders with low familial recurrence is hardly possible. This applies, for example, to RTT being mainly caused by a rare, spontaneous genetic mutation (Amir et al. 1999). Participants must be sought out and recruited at a time at which their developmental outcome is already known, i.e., a diagnosis has already been made. Data are collected retrospectively, i.e., ‘in the past’, for example, via parents’ associations, social networks, email blasts, or by cooperating with clinical expertise centers.

### In the Past

In most cases, retrospectively collected data including audio of preverbal behavior are home videos provided by the participants’ families (e.g., Boterberg et al. 2019; Einspieler and Marschik 2019; Marschik and Einspieler 2011; Roche et al. 2018). Audio–video recordings were typically taken by the participants’ parents, who were not aware of their children’s potential later diagnosis. Usually, they used standard video cameras or, in recent years, to a greater extent smartphones. On the basis of home videos, participants can be retrospectively studied in their natural environment during everyday situations, such as interactive playing, during typical family routines, such as bathing, diaper-changing, or feeding situations, or during special events, such as familial celebrations. However, neither recording quality nor recording setting are a priori controlled study design criteria. Inhomogeneous recording formats and microphone/camera positions, in combination with uncontrollable background noise conditions, cause a large acoustic variation within a home video dataset. For example, our Graz University Audiovisual Research Database for the Interdisciplinary Analysis of Neurodevelopment (GUARDIAN), contains home video data with a total runtime of several months. The earliest clips were taken in the 1960s. Their original video formats range from analogue standards, such as Super 8 or VHS, to state-of-the-art digital standards (Pokorny et al. 2016b). Studies based on preverbal data collected ‘in the past’ typically lack of standardization and reproducibility, and thus have limited comparability to other studies. Furthermore, the interactive setting that potentially influences an infant’s preverbal behavior can only be partly evaluated as other persons or interactive (media) devices might be out of the recording device’s range, e.g., out of the video camera’s view. Another limitation is the potential absence of a specific behavior of interest within a collected dataset, such as the production of specific preverbal vocalization types. Moreover, the exact age of the recorded participant in a clip is often unknown and can only be roughly estimated. Regarding documentation of participant outcome, different diagnostic assessments are used (e.g., ADOS, ABC, or CARS for individuals with ASD) and often varying details on symptom severity and comorbidities are available. Nevertheless, data collection ‘in the past’ offers a unique opportunity to ‘listen back’ to preverbal phenomena. For the moment, it still represents one of the best strategies we have for studying the preverbal development of individuals with developmental disorders with a late clinical manifestation (e.g., Adrien et al. 1993; Crais et al. 2006; Marschik and Einspieler 2011; Palomo et al. 2006; Saint-Georges et al. 2010).

### In the Lab

The prospective collection of preverbal data in a laboratory allows for controllable acoustic room conditions and a careful a priori definition of the optimal recording device/quality and the recording setting. The recording procedure is, thus, reproducible and enables the best possible data comparability. Moreover, participants can be invited according to a predefined study paradigm and appropriate time plan to collect preverbal data from exactly defined age windows. Exemplarily, the CRIED (Cry Recognition In Early Development) database of 5587 mood-annotated preverbal vocalizations from 20 individuals (Schuller et al. 2018) was collected at the Medical University of Graz, Austria, in the framework of a longitudinal study on typical neuro-functional and neuro-behavioral changes in early infancy (Marschik et al. 2017). Participants were recorded 7 times on a bi-weekly basis with the first session at 4 weeks ± 2 days and the last session at 16 weeks ± 2 days post-term age. During the sessions, the infants were lying awake in supine position in a cot with various recording devices including microphones positioned around (Marschik et al. 2017). Data collection in the lab generally implies that participants are recorded in an artificial environment. A popular laboratory setting in which some aspects of the participants’ natural environment are simulated, is a parent–child-interaction setting. In such a setting, parents are, e.g., instructed to play with their children with a predefined set of toys in a standardized recording room as they would do at home (e.g., Pokorny et al. 2017).

Apart from lab recordings targeting the collection of infant vocalizations, data originally recorded for other reasons can also represent a valuable source for compiling preverbal behavior analysis datasets. Such data could be, for example, audio–video recordings of developmental assessments routinely made for documentation or evaluation purposes.

### In the Wild

The prospective collection of preverbal data under real-world conditions, i.e., in the participants’ natural environment, permits predefining a desired recording quality. The only limiting requirement is that the recording device has to be placed ‘in the wild’. This plays a role for the selection of an optimal device/microphone type, e.g., with respect to its dimensions or power supply. Typically, camcorders are used for audio–video data collection ‘in the wild’, while mobile wave recorders are used for audio data collection only. Alternatively, nowadays data can be easily collected via parental smartphones. In recent years, naturalistic data for studies on preverbal typical and atypical development have been frequently collected with the LENA^®^ (Language Environment Analysis; https://www.lena.org [as of 5 April 2019]; Xu et al. 2008a, b) system (e.g., Oller et al. 2010; Swanson et al. 2018). The LENA^®^ system enables the long-term recording of infants’ vocalizations by means of a child-safe audio recording device attached to a vest (Xu et al. 2008a, b). Except for the position of the recording device, recording setting parameters, such as position, posture, and activity of the participant, are uncontrolled. Acoustic background noise conditions can only be marginally controlled, e.g., by giving parents respective instructions. All things considered, comparability of data collected ‘in the wild’ is very limited. Studies are only partially reproducible.

Of course, combinations of different data collection strategies are conceivable, such as data collection in the lab around a specific first age window of interest and data collection ‘in the wild’ around a specific second age window of interest. Besides the actual recording of preverbal data, both prospective approaches, i.e., data collection in the lab and ‘in the wild’, offer an easy way to acquire relevant additional information on the participants from the caregivers without running the risk of memory bias (e.g., Marschik 2014; Ozonoff et al. 2011b; Palomo et al. 2006; Zhang et al. 2017). However, in prospective approaches the participants’ developmental outcomes are not yet known at the time of data collection and have to be evaluated in follow-up assessments.

## Data Representation

For intelligent audio analysis purposes, an approriate acoustic representation has to be derived from the collected audio data that contain preverbal behavior. This process can be regarded as acoustic behavior modeling with the goal to gain distinct information characterizing a preverbal phenomenon. The appropriateness of a specific data representation highly depends on the intended type of subsequent analysis or learning task. Basically, the representation of acoustic data can be either based on predefined features (“Feature-Based representation” section) or automatically learned in context of a specific task (“Representation learning” section). However, some preprocessing steps need to be carried out before.

### Preprocessing

First of all, preverbal behavior has to be identified and segmented within the recorded audio material. Segmentation describes the process of setting boundaries around identified preverbal behavior in order to create meaningful analyzable units. Depending on the intended analysis, classification task, or target application, meaningful units can be, for example, single phones, but also extensive phrases. In many cases, preverbal behavior is segmented into utterances that are denominated as preverbal vocalizations and generally lie in between the duration of a phone and a phrase (Lynch et al. 1995; Nathani and Oller 2001). There are two common procedures for utterance segmentation. The first procedure is based on setting segment boundaries at vocal pauses, i.e., phases without vocal activity, exceeding a predefined pause duration (e.g., Nathani and Oller 2001). Considering the physiological process of voice production as being linked to respiratory activity, the second segmentation procedure relies on the condition that each vocalization has to be assigned to a distinct vocal breathing group. Segment boundaries, thus, coincide with phases of ingressive breathing (e.g., Nathani and Oller 2001; Oller and Lynch 1992).

The LENA^®^ system, for example, provides fully automatic vocalization segmentation and diarization alongside recorded audio. Diarization in this context means that the system automatically indicates if a vocalization was most probably produced by the infant of interest or, for example, by a caregiver (Xu et al. 2008a). However, automatic segmentation and diarization is prone to errors, especially if the audio material includes acoustic disturbances as presumed for data collected ‘in the wild’ or ‘in the past’ (Pokorny et al. 2016b). Therefore, preverbal data segmentation and diarization is still often done manually or at least semi-automated. In a semi-automated procedure, automatically created segments are manually checked for correctness. However, false negatives are missed out.

Another essential preprocessing step is the annotation of preverbal behavior. Each included segment needs to be labeled with study-relevant meta-information. On the one hand, there are behavior-independent variables implicating that the labels stay the same for all preverbal behaviors of, e.g., one and the same participant in one and the same recorded clip. Examples are participant-dependent variables, such as gender, family language, medical condition/later diagnosis, or age at the time of recording. In addition, data collected in non-standardized settings, i.e., ‘in the wild’ or ‘in the past’, usually need to be annotated for all factors that might have acoustically or physically influenced the recorded preverbal behavior, such as scene type (playtime, bathtime, mealtime, etc.), location (indoor vs. outdoor), presence and type of background noise, interactive setting, participant posture, participant physical restriction, or use of a pacifier. On the other hand, there are behavior-dependent variables, such as the number of syllabic elements within an infant vocalization, or the preverbal vocalization type based on a vocalization classification scheme, e.g., the Stark Assessment of Early Vocal Development-Revised (SAEVD-R; Nathani et al. 2006).

An optional signal-related data preprocessing step for subsequent intelligent audio analysis purposes is audio normalization, i.e., setting the audio signal’s maximum amplitude to a defined level (e.g., Eyben et al. 2013b, c; Schuller et al. 2016). This is done to guarantee a comparable amplitude range across all included raw audio data of a dataset and can especially be beneficial when analyzing sets of data that were recorded with different recording devices at different recording levels. Dependent on the nature of the collected raw material, audio normalization is either done segment-wisely, i.e., in separate for each vocalization, or globally prior to the segmentation process.

### Feature-Based Representation

The traditional audio representation for subsequent learning tasks builds upon the extraction of acoustic features from the collected and preprocessed audio data. A segment of preverbal behavior is transformed into a single multidimensional feature vector or a chronological series of feature vectors acoustically modeling the behavior‘s trajectory. Acoustic features are mathematical descriptors defined on the basis of a priori expert knowledge with the intention to characterize the content of an audio signal in a compact, informative, but preferably non-redundant way (Schuller 2013). Optimal acoustic features for preverbal behavior modeling or speech applications in general (e.g., Schuller and Batliner 2014) might differ from optimal acoustic features for other applications. Basically, the transformation of audio into a meaningful feature representation implies a reduction of information (Schuller 2013). The number of features typically extracted for subsequent speech-related learning tasks varies from less than 100 (e.g., Deng et al. 2017; Eyben et al. 2016) to several thousand (e.g., Schuller et al. 2013). Dependent on the applied machine learning algorithm, large feature vectors are either directly used as input representation for learning/classification, or reduced to an optimized feature subset by means of feature selection algorithms (Cai et al. 2018). ‘Optimized’ in this context means that features that do not or only marginally contain information relevant for the intended learning/classification task are identified and discarded.

Natural audio signals are usually time variant, i.e., they change over time (Deller et al. 1993). This also holds true for recorded preverbal behavior. Consequently, the extraction of acoustic features is usually carried out on the basis of short, window-function-weighted, overlapping time frames. Within each time frame, audio information is considered to be quasi-stationary (Deller et al. 1993). Features derived from this first level of signal sub-sampling are denominated as low-level descriptors (LLDs; Schuller 2013; Schuller and Batliner 2014).

There are a number of well-established acoustic LLDs, such as the F0. However, different mathematical and methodological ways of extracting one and the same feature exist. Moreover, the exact sequence of calculation steps including all set adjustments for deriving a specific feature from an audio signal is usually not specified in publications. This hampers the comparability of absolute feature values reported across different studies. Therefore, open-source feature extraction tools steadily grow in popularity in the research community. Such tools allow for reproducible feature calculation throughout different labs around the world. Furthermore, standard feature sets can be provided. A popular open-source feature extraction tool kit is openSMILE by audEERING™ GmbH (https://audeering.com/technology/opensmile/ [as of 8 April 2019]). openSMILE is based on C++, enables feature extraction both offline and in real-time, and comes along with standard feature sets well-proven for various application areas (Eyben et al. 2010, 2013a).

The so far most comprehensive standard feature set for openSMILE is the ComParE set. It is widely known, as it represented the official baseline feature set of the 2013–2019 Computational Paralinguistics ChallengEs (e.g., Schuller et al. 2013, 2018, 2019) carried out in connection with the Annual Conferences of the International Speech Communication Association (INTERSPEECH conferences). The ComParE set comprises 6373 acoustic supra-segmental features, so-called higher-level descriptors (HLDs). These are statistical functionals computed for the trajectories of a wide range of acoustic time-, energy-, and/or spectral/cepstral-based LLDs as well as their derivatives (Schuller et al. 2013). In contrast, the most current standard feature set for openSMILE is the Geneva Minimalistic Acoustic Parameter Set (GeMAPS). It was launched in 2016 by Eyben et al. (2016) and represents a comparatively small set of only 62 frequency-, energy-, and spectral-related features. Its extended version, the eGeMAPS, contains 26 additional frequency- and spectral-related descriptors summing up to a total of 88 features. The features of the eGeMAPS were carefully selected based on (a) their theoretical and practical relevance for automatic voice analysis applications, including clinical applications, and (b) their proven value in previous studies in the related fields (Eyben et al. 2016). By default, LLD trajectories for both the ComParE set and the eGeMAPS are extracted on the basis of overlapping time frames of 60 ms at a step size of 10 ms. LLD contours are smoothed over an interval of three frames (unvoiced-voiced transitions in selected LLD contours excepted). HLDs are then calculated for the smoothed LLD contours of the entire input segment resulting in exactly one vector of 6373 or 88 feature values per segmented preverbal behavior, respectively. A data representation like this is suited for static learners. For dynamic learners, i.e., algorithms operating on the acoustic content’s variations over time, HLD trajectories have to be calculated per segment on an appropriate time basis, or the LLD contours are used for representing each segmented behavior.

Both the ComParE set and the eGeMAPS have already successfully been applied for demonstrating the feasibility of an automatic preverbal vocalization-based differentiation between typically developing (TD) individuals and individuals with late detected developmental disorders (Pokorny et al. 2016a, 2017). Pokorny et al. (2016a) extracted the ComParE features from 4678 retrospectively collected preverbal vocalizations of four TD infants and four infants later diagnosed with RTT. A promising mean unweighted accuracy of 76.5% was achieved in the binary vocalization classification paradigm RTT versus TD using linear kernel support vector machines as classifier in a four-fold leave-one-speaker-pair-out cross-validation scheme. The study by Pokorny et al. (2017) built upon data collected at a participants’ age of 10 months in a semi-standardized parent–child-interaction setting within the prospective ASD high-risk protocol EASE (Early Autism Sweden; http://www.earlyautism.se [as of 3 April 2019]). Both the eGeMAPS features and the LLDs of the ComParE set were extracted from 684 preverbal vocalizations of 10 TD individuals and 10 individuals later diagnosed with ASD. The eGeMAPS features were used as data representation for static classification by means of linear kernel support vector machines. In contrast, dynamic modeling was investigated by applying a neural network classifier on the basis of the LLDs of the ComParE set. Three-fold cross-validation was carried out for performance evaluation. Either approach led to 15 of 20 infants correctly assigned to group ASD or TD.

A popular data representation that builds upon extracted LLD or HLD contours is the Bag-of-Audio-Words (BoAW) representation (e.g., Lim et al. 2015; Pancoast and Akbacak 2014; Pokorny et al. 2015; Schmitt et al. 2016). It relies on the quantization of input feature trajectories according to a learned codebook. Finally, data are represented as histograms of the previously generated sequence of ‘audio words’. A recently introduced open-source tool kit for the extraction of Bo(A)W from arbitrary, multidimensional input feature trajectories is openXBOW (https://github.com/openXBOW [as of 9 April 2019]; Schmitt and Schuller 2017). Since 2017, openXBOW has been used in addition to openSMILE as official baseline feature extractor of the annual INTERSPEECH ComParE Challenges (e.g., Schuller et al. 2017). Within the INTERSPEECH 2018 ComParE Challenge (Schuller et al. 2018), openXBOW was for the first time used to generate BoAW from preverbal behavior: In the Crying Sub-Challenge, vocalizations of the CRIED database had to be automatically told apart according to three mood-related vocalization classes, namely (1) neutral/positive sounds, (2) crying sounds, and (3) fussing sounds, which can be regarded as transition behavior between (1) and (2) (Schuller et al. 2018).

Further feature-related data representations have been recently used for intelligent audio analysis applications, such as Bag-of-Context-Aware-Words (e.g., Han et al. 2018). However, to the best of our knowledge, such representations have played a minor role for the acoustic modeling of preverbal behavior in context of typical or atypical development so far.

To meet the requirements of some classifiers (Bishop 2006), a common feature post-processing step is feature normalization or standardization. This means that feature values are rescaled to be located within a defined interval, such as [0, 1], or to have zero mean and unit variance, respectively. Feature normalization/standardization can be carried out globally, i.e., based on all instances/vocalizations of a dataset. Alternatively, it can be done in separate for semantic instance sub-partitions, e.g., participant/infant-wisely.

### Representation Learning

In contrast to feature-based modeling, representation learning seeks for automatically deriving a data representation that makes subsequent learning tasks easier (Goodfellow et al. 2016). Thereby, a trade-off between reaching beneficial properties for subsequent classification and keeping as much information about the input signal as possible, has to be found. Representation learning can be (a) supervised, i.e., based on class-labeled data, (b) unsupervised, i.e., based on unlabeled data, or (c) semi-supervised, i.e., based on a usually small amount of labeled and a usually large amount of unlabeled data (Goodfellow et al. 2016). Following general directions in machine learning, especially deep representation learning has received increasing attention (Bengio et al. 2013) and proven powerful in a number of intelligent audio analysis scenarios in recent years. An open-source tool kit for deep unsupervised representation learning in the audio domain was introduced by Freitag et al. (2017): AUDEEP is a Python tool based on the widely used open-source machine learning library TENSORFLOW™ (https://www.tensorflow.org [as of 9 April 2019]; Abadi et al. 2016). It allows for learning data representations from audio time series by means of a recurrent sequence-to-sequence autoencoder approach (Freitag et al. 2017). Complementing the brute-force feature extraction tools openSMILE and openXBOW, in 2018 AUDEEP was elected as open-source representation learning tool kit for official baseline evaluation within the ongoing series of INTERSPEECH ComParE Challenges (Schuller et al. 2018, 2019). Thus, in that year, AUDEEP was initially applied to learn representations from preverbal data. Within the Crying Sub-Challenge of the 2018 INTERSPEECH ComParE Challenge (Schuller et al. 2018), representations were automatically derived from Mel-scale spectrograms generated for the vocalizations of the CRIED database. Similarly, based on input spectrograms, Cummins et al. (2017) proposed a deep spectrum feature representation for emotional speech recognition in children. Here, spectrograms are passed through a deep image classification convolutional neural network. The intended representation is then derived from the activations of the last fully-connected layer of the network (Cummins et al. 2017). However, empirical studies using this approach for modeling preverbal data are still outstanding. (Deep) representation learning in general, still describes a very young methodology for processing preverbal data of typical and atypical development.

A method in which the optimal data representation and the subsequent classifier are learned simultaneously from the audio signal is end-to-end learning. End-to-end learning models were also applied to the CRIED database in the framework of the 2018 INTERSPEECH ComParE Challenge (Schuller et al. 2018). However, this method is not further treated here, as in end-to-end learning representation learning can not be regarded independently from the subsequent classification algorithm.

## Discussion

Preverbal human development has been a popular research field for almost 40 years (e.g., Nathani et al. 2006; Oller 1980, 2000; Papoušek 1994; Stark 1980). Researchers and clinicians from different disciplines, such as linguists, neurologists, pediatricians, psychologists, physiologists, speech-language therapists, and engineers, have synergetically characterized preverbal phenomena in typical and atypical development over the years. Objective empirical investigations of preverbal behavior have, however, always required the audio recording of preverbal behavior as well as the recorded behavior’s meaningful representation for subsequent analyses. A number of strategies for the collection and representation of preverbal data exist. Some of these strategies have even been successfully applied in intelligent audio analysis paradigms on infants with late detected developmental disorders testing automatic earlier identification (e.g., Oller et al. 2010; Orlandi et al. 2012; Pokorny et al. 2016a, 2017; Xu et al. 2009). The question of what makes collection and representation of preverbal data efficient, has to be answered in the context of the specific learning task and its target area of application.

Regarding data collection, efficiency might be quantified as the proportion between (a) quality and quantity of collected preverbal data, and (b) collection efforts in time and money. In this context, data quality refers to recording quality on the one hand, and to influences on the recorded behavior of interest on the other hand. Quantity refers to preverbal behavior of interest within a recording, not to the overall recording duration. Both quality and quantity depend on recording setting and environment. The general demand of recording participants within their natural environment in order not to get their behavior influenced by the experimental setup should be considered as a function of age. Human vocal behavior has been discussed to be endogenously generated in early infancy by specific neural networks in the brain stem (Barlow et al. 2010), so-called central pattern generators (e.g., Barlow and Estep 2006; Fénelon et al. 1998). We thus might assume that an artificial laboratory environment will hardly influence the preverbal behavior of newborns or participants in the early postnatal period. When trying to collect representative data of participants’ natural behavior during late infancy, the recording environment may then play a crucial role. However, apart from age-related aspects and the limitation that specific data collection strategies are not suitable for specific participant groups, e.g., prospective approaches for participants with rare late detected developmental disorders, each data collection strategy has its strengths and weaknesses with respect to the above discussed efficiency criteria (Fig. 1). A careful hypotheses-oriented a priori study design planning under consideration of available time and budget may guarantee maximum efficiency in data collection.

‘Efficient’ in representing preverbal data may mean that collected data are best possibly reduced to specific information that is needed to reach high data interpretability in subsequent analyses or high performance in subsequent learning tasks. Of course, required processing power and computing time have to be taken into account, especially in the context of potential real-time applications with devices of limited computing capacities, such as smartphones. For example, when using a large brute-force feature set and a classifier that is not prone to overfitting, a feature selection procedure can be left out. However, the choice of efficient data representation not only depends on a subsequent analysis or learning task. It also depends on the nature of collected data and, thus, on the applied data collection strategy. For example, audio normalization as a representation pre-processing step might not make sense for recordings that include background noise events exceeding the level of preverbal behaviors.

In Table 1, we present recognition results that were achieved in a binary preverbal vocalization classification paradigm RTT versus TD. Data for this experiment were collected ‘in the past’. 3502 preverbal vocalizations were extracted from home video recordings of 3 TD individuals and 3 individuals later diagnosed with RTT. The recordings were made in the individuals’ second half year of life, respectively. Some alternatives with regard to data representation were tried out, namely (1) segment-wise audio normalization yes versus no, (2) ComParE set versus eGeMAPS, and (3) infant-wise versus global feature normalization. Then, we trained, optimized, and evaluated linear kernel support vector machines in a three-fold leave-one-speaker-pair-out cross-validation scheme. Finally, we stored the predictions of each iteration and calculated the unweighted average recall (UAR) for the whole dataset. The best performance—a UAR of .879—was achieved when (1) processing normalized audio segments, (2) using the eGeMAPS, and (3) applying infant-wise feature normalization. Generally, there were only marginal differences between the scenarios with and without audio normalization. However, on average the scenarios without audio normalization reached slightly better results. This might be due to the nature of data used for this experiment—home video data involving everyday background noise. Segment-wise audio normalization of background noise-prone recordings may have caused disadvantageous level imbalances between segments with background noise exceeding the level of the preverbal behavior of interest and segments without background noise. The greatest effect in this experiment was related to the choice of feature set. The eGeMAPS clearly outperformed the ComParE set here. Finally, infant-wise feature normalization turned out more beneficial compared to global feature normalization.

**Table 1:** Comparison of system configurations regarding audio normalization, used feature set, and feature normalization strategy (infant-wise: normalization in separate for all vocalizations of an infant, respectively; global: normalization over all instances of the dataset; see also last paragraph of “Feature-based representation” section) by means of the unweighted average recall (UAR) achieved in a binary vocalization classification paradigm RTT versus TD

Audio normalization	Feature set	Feature normalization	UAR_RTT vs. TD_
–	ComParE	infant-wise	.356
–	ComParE	global	.399
–	eGeMAPS	infant-wise	.832
–	eGeMAPS	global	.674
✓	ComParE	infant-wise	.372
✓	ComParE	global	.281
✓	eGeMAPS	infant-wise	.879
✓	eGeMAPS	global	.535

UAR values are rounded to three decimal places

*ComParE* Computational Paralinguistics ChallengE (feature set), *eGeMAPS* extended Geneva Minimalistic Acoustic Parameter Set, *RTT* Rett syndrome, *TD* typical development, ✓ applied, – not applied

For answering the question of better to apply a brute-force feature set, such as the eGeMAPS, or features automatically learned from data, it should be considered that deep representation learning methods usually require very large datasets (Bengio et al. 2013) and that automatically learned features are hardly acoustically or voice-physiologically interpretable anymore. The latter aspect might be relevant if clinical conclusions shall be drawn from the data representation.

In this paper, we provided an overview and discussed current strategies for collecting and acoustically representing preverbal data for intelligent audio analysis paradigms on typical and atypical early development. A special focus was given to methodological requirements and limitations for studies on the preverbal behavior of individuals with late detected developmental disorders. With the rising age of deep learning, significant advancements have been made in intelligent audio analysis in recent years. However, the application of state-of-the-art audio processing and machine learning methods for preverbal behavior analysis, especially in a clinical context of automatically differentiating orthology and pathology, has only just started. Progress in the acoustic modeling of preverbal data over the coming years is thus warranted. In addition, future collection of preverbal data will most probably be influenced by industrial and social trends, such as by the combination of smartphone development and people’s increasing affinity for self-tracking during daily life (e.g., Lupton and Smith 2018). Consumers, thus also parents, get more and more equipped with affordable powerful tools facilitating an extensive documentation of their own, but also of their children’s lives. Masses of new data will thereby be generated. In a couple of years, these data will be available for being collected ‘in the past’.
